# The Transcriptional Co-factor IRF2BP2: A New Player in Tumor Development and Microenvironment

**DOI:** 10.3389/fcell.2021.655307

**Published:** 2021-04-29

**Authors:** Tatiane P. Pastor, Barbara C. Peixoto, João P. B. Viola

**Affiliations:** Program of Immunology and Tumor Biology, Brazilian National Cancer Institute (INCA), Rio de Janeiro, Brazil

**Keywords:** IRF2BP2, tumor suppressor, transcriptional regulation, tumor development, tumor microenvironment

## Abstract

Interferon regulatory factor 2-binding protein 2 (IRF2BP2) encodes a member of the IRF2BP family of transcriptional regulators, which includes IRF2BP1, IRF2BP2, and IRF2BPL (EAP1). IRF2BP2 was initially identified as a transcriptional corepressor that was dependent on Interferon regulatory factor-2 (IRF-2). The IRF2BP2 protein is found in different organisms and has been described as ubiquitously expressed in normal and tumor cells and tissues, indicating a possible role for this transcriptional cofactor in different cell signaling pathways. Recent data suggest the involvement of IRF2BP2 in the regulation of several cellular functions, such as the cell cycle, cell death, angiogenesis, inflammation and immune response, thereby contributing to physiological cell homeostasis. However, an imbalance in IRF2BP2 function may be related to the pathophysiology of cancer. Some studies have shown the association of IRF2BP2 expression in hematopoietic and solid tumors through mechanisms based on gene fusion and point mutations in gene coding sequences, and although the biological functions of these types of hybrid and mutant proteins are not yet known, they are thought to be involved in an increase in the likelihood of tumor development. In this review, we address the possible involvement of IRF2BP2 in tumorigenesis through its regulation of important pathways involved in tumor development.

## Introduction

The interferon regulatory factor 2-binding protein (IRF2BP) family of transcription regulators includes IRF2BP1, IRF2BP2, and IRF2BPL (also known as EAPI – enhanced at puberty 1), all three of which are nuclear proteins. IRF2BP2 proteins were first identified *via* a yeast two-hybrid assay as nuclear transcription corepressors of interferon regulatory factor-2 (IRF-2) that inhibit both enhancer-activated and basal transcription. More recently, IRF2BP2 was identified as a transcriptional repressor in several other biological contexts that do not require IRF-2 participation, suggesting that IRF2BP2 has IRF-2-independent functions (Childs and Goodbourn, 2003).

Interferon regulatory factor 2-binding protein 2 is encoded by a gene located on chromosome 1q42.3 in humans and has two exons producing three alternatively spliced proteins, IRF2BP2A with 587 amino acids, IRF2BP2B with 571 amino acids and IRF2BP2C with 163 amino acids. IRF2BP2 isoforms A and B share high identity and display two conserved regions: a zinc finger domain at the N-terminus, which is missing in IRF2BP2 isoform C, and a CH3C4 real interesting new gene (RING) domain at the C-terminus. The function of these domains in the IRF2BP2 isoforms is not fully understood, but both domains have been described as being important for their ability to mediate interactions between different proteins. Between these domains, there is a region formed by arginine and lysine residues (RKRK), with the nuclear localization signal (NLS), which is conserved among family members (Carneiro et al., 2011; Teng et al., 2011). Teng et al. (2011) showed that phosphorylation of Ser360 near the NLS, as identified in IRF2BP2 isoform A, favors IRF2BP2 nuclear localization (Teng et al., 2011).

The IRF2BP2 protein is observed in different organisms and is ubiquitously expressed in different normal and tumor cells and tissues, as determined through analyses of the transcriptome and proteome, as related by Fagerberg et al. (2014), that this transcriptional cofactor plays possible roles in different cell signaling pathways.

## Biological Functions of IRF2BP2

Despite being identified as a transcriptional partner of IRF-2, the IRF2BP2 protein is also observed in organisms lacking this transcription factor, showing that this protein has IRF-2-independent functions through interactions with other partners involved in transcriptional regulation (Childs and Goodbourn, 2003). Although the majority of reports available in the literature propose a role for IRF2BP2 as a repressor in the regulation of diverse genes, some studies have shown that IRF2BP2 may also act as a positive regulator of gene expression (Teng et al., 2010; Chen et al., 2015; Cruz et al., 2017). Although IRF2BP2 is an important regulator of gene expression, the mechanisms by which IRF2BP2 mediates its repression or induction have not been established and may involve interactions with different proteins that vary according to the context.

**Figure 1:**
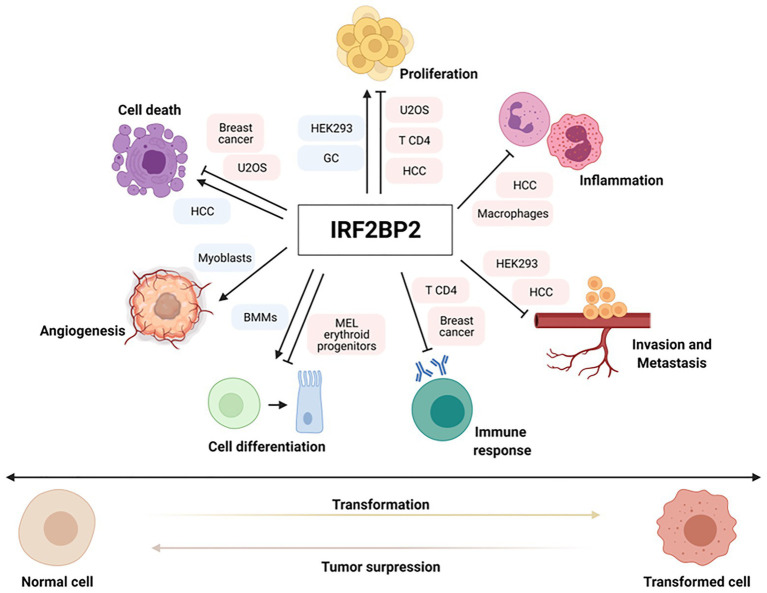
Important pathways for tumor development are regulated by interferon regulatory factor 2-binding protein 2 (IRF2BP2). IRF2BP2 proteins are involved in regulating positive or negative expression of genes related to various hallmarks of oncogenesis, such as cell proliferation, apoptosis, cell differentiation, angiogenesis, immune response, inflammation and invasion/metastasis in normal and cancer setting. Boxes indicate normal and tumor cells where IRF2BP2 protein acts as a positive (blue) or negative (red) regulator of different cell functions.

Interferon regulatory factor 2-binding protein 2 is extensively involved in regulating gene expression and other biological processes, including metabolic syndrome, nervous diseases, immunodeficiency disorders, and cancer. Recent data suggest the participation of IRF2BP2 in controlling various cellular functions, such as cell proliferation (Koeppel et al., 2009; Secca et al., 2016; Manjur et al., 2019; Yao et al., 2019; Feng et al., 2020), apoptosis (Koeppel et al., 2009; Tinnikov et al., 2009; Yeung et al., 2011; Feng et al., 2020; Li et al., 2020), angiogenesis (Teng et al., 2010), cell migration (Manjur et al., 2019; Feng et al., 2020), cell differentiation (Stadhouders et al., 2015; Kim et al., 2019; Wang et al., 2020a), inflammation (Chen et al., 2015; Cruz et al., 2017; Hari et al., 2017; Feng et al., 2020; Li et al., 2020), and immune response (Blotta et al., 2009; Carneiro et al., 2011; Soliman et al., 2014; Dorand et al., 2016; Secca et al., 2016; Wu et al., 2019), contributing to physiological homeostasis and the hallmarks of oncogenesis (Figure 1). Different studies have shown the association of IRF2BP2 expression in hematopoietic malignancies and solid tumors, such as breast cancer, leukemia and chondrosarcoma, through mechanisms of gene fusion and point mutations in gene coding sequences, but its exact role and the regulatory mechanism in cancers have not been explored. This review focuses on the involvement of IRF2BP2 in tumorigenesis through the regulation of important pathways in tumor development.

## IRF2BP2 in Cell Proliferation

Tissue homeostasis is characterized by a delicate balance between cell proliferation and death, and several signaling mechanisms determine whether a particular cell remains quiescent, proliferates or dies. Therefore, proliferation is an important part of cancer development and progression and manifests with altered expression and/or activity of cell cycle-related proteins (Malumbres and Barbacid, 2001; Caglar and Avci, 2020).

Several studies have demonstrated the important function of IRF2BP2 in controlling the cell cycle and cell proliferation. Koeppel et al. (2009) identified *IRF2BP2* as a novel gene directly targeted by the p53 protein, influencing cell fate. In this study, it was shown that in response to genotoxic stress, p53 binds to an upstream site in the *IRF2BP2* promoter and activates its expression. Consequently, IRF2BP2 overexpression represses the p53-mediated transactivation of the p21 gene and can induce changes in the cell cycle of living cells, mainly changing the number of cells in the S-phase population, which decreases after actinomycin D (Act. D) treatment, favoring cell cycle arrest and survival of U2OS osteosarcoma cells (Koeppel et al., 2009).

Viola’s study demonstrated that the ectopic overexpression of IRF2BP2 in primary CD4 T cells leads to a decrease in cell proliferation upon activation and reduces the expression of the activation markers CD69 and CD25 *in vitro*. In addition, CD4 T cells overexpressing IRF2BP2 were transferred to sublethally irradiated C57/BL6 recipient mice to observe their expansion *in vivo*. The lymphocytes transduced with *IRF2BP2* showed reduced cell expansion, indicating that this protein had a negative effect on the survival of these cells (Secca et al., 2016).

Manjur et al. (2019) analyzed the role of IRF2BP2 in the regulation of glucocorticoid (GC) signaling. For this study, IRF2BP2 was silenced by siRNA in HEK293-GR cells, and gene expression was analyzed using RNA-seq. IRF2BP2 modulated the transcription of approximately one half of the GC-responsive genes, leading to a reduction in the proliferation of HEK293-GR cells. To investigate the predicted effect of IRF2BP2 silencing on HEK293-GR proliferation, a live-cell imaging assay was performed, and the results confirmed those obtained *via* RNA-seq, suggesting that IRF2BP2 acts as a positive regulator of cell proliferation and is part of the GR transcription regulatory complex and functions as a novel coregulator of a subset of target genes associated with glucocorticoid functions (Manjur et al., 2019).

Another study found that IRF2BP2 is a direct target of components in the Hippo pathway, an evolutionarily conserved regulator of cell growth. Interestingly, IRF2BP2 overexpression in two hepatocellular carcinoma (HCC) cell lines (HepG2 and Huh7 cells) negatively regulated YAP activity and decreased the expression levels of YAP target genes. An MTT analysis of cell proliferation and a colony formation assay showed that cells stably overexpressing IRF2BP2 exhibited suppressed growth and reduced colony formation ability, respectively. In addition, HCC cells overexpressing IRF2BP2 were inoculated in the flanks of nude mice, and interestingly, IRF2BP2 overexpression failed to lead to tumors formation, compared to their formation in control groups. To confirm these results, the authors examined the loss-of-function phenotypes of IRF2BP2 in HepG2 cells. IRF2BP2-deficient (IRF2BP2−/−) cells were established by the CRISPR-CAS9 technique. IRF2BP2 depletion led to enhanced cell growth and colony formation and promoted HepG2 xenograft tumor growth. Taken together, these results showed that IRF2BP2 exhibits tumor-suppressor activity in the liver, inhibiting liver cancer cell growth and tumor formation (Feng et al., 2020).

On the other hand, recent study showed an opposite effect of IRF2BP2 on cell proliferation. MTT and colony formation assay results suggested that knocking down IRF2BP2 by siRNAs significantly decreased the proliferation and colony formation rates of human gastric (GC) cell lines. Additionally, GC cells expressing IRF2BP2 shRNA were inoculated subcutaneously in mice to construct xenograft models, and the results showed that knocking down IRF2BP2 significantly inhibited tumor growth. This study demonstrated that IRF2BP2 knockdown decreased cell proliferation by inhibiting the binding of TEAD4 to YAP1, which then could no longer promote the transcription of genes downstream of YAP1, such as CTCF, an important oncogene related to the promotion of GC cell proliferation (Yao et al., 2019). It important to note that, the differences in cellular proliferation phenotypes observed in previous studies may be associated with the different interaction partners and/or target genes of the IRF2BP2, which may be involved with the type of cell and tumor involved.

Furthermore, Barysch et al. (2021) investigated the functional consequences of endothelial growth factor (EGF)-dependent deSUMOylation of IRF2BP2 protein. They demonstrated that IRF2BP2 protein lost SUMO upon EGF treatment in HeLa cells and this deSUMOylation is very transient. Two SUMO motifs that were conserved within the family IRF2BP have been identified, one of which is located in the C-terminal region. IRF2BP2 knockdown in HeLa cells led to an upregulation of EGFR which may contribute to enhanced proliferation in response to EGF, a potent regulator of cellular growth as well as in cancer progression (Barysch et al., 2021).

## IRF2BP2 in the Regulation of Apoptosis

To prevent cell proliferation, programmed cell death processes follow ordered sequences of events that lead to cell death. The process of programmed cell death, or apoptosis, is generally characterized by distinct morphological characteristics and is considered a vital component of various processes, including normal cell turnover. However, inappropriate apoptosis is a factor in many human conditions, including different types of cancer (Elmore, 2007).

Koeppel et al. (2009) showed that IRF2BP2, in addition to inhibiting the expression of the p21 gene, also acts as a repressor of p53-mediated transactivation of the BAX gene, a potent proapoptotic gene. U2OS cells were transfected with IRF2BP2 and treated with doxorubicin to induce apoptosis. In the *IRF2BP2*-transfected cells, treatment with doxorubicin failed to induce an increase in the population of apoptotic cells (as indicated by the sub-G1 population). To investigate the function of IRF2BP2 in apoptosis under physiological conditions, endogenous levels of IRF2BP2 were downregulated by siRNAs in U2OS cells. Upon the treatment with Act. D and doxorubicin, the IRF2BP2-knockdown cells showed high levels of activated Caspase 3. According to the authors, these results suggest that IRF2BP2 is an important factor in the determination between cell survival and cell death (Koeppel et al., 2009).

Interferon regulatory factor 2-binding protein 2 isoform A was identified in a yeast double-hybrid assay, revealing its interaction with NRIF3, which is a proapoptotic factor in breast cancer cells. In this study, different breast cancer cell lines were transfected with a siRNA that targets IRF2BP2A mRNA, and all of the cell lines exhibited an increased apoptosis rate, suggesting that IRF2BP2A acted to selectively repress proapoptotic genes and thus functioned as an anti-apoptotic factor (Tinnikov et al., 2009). In 2011, the same group showed that IRF2BP2A performs this function by suppressing the proapoptotic gene FASTKD2. Through chromatin immunoprecipitation and mass spectrometry analyses, the IRF2BP2A protein was identified as one of the members of a large protein complex that also includes IRF2BP1 and EAP1 proteins. These proteins interact through their C4 zinc finger domains and bind to DNA near the transcription region of the FASTKD2 gene, repressing its transactivation and thus selectively modulating the cell survival and apoptosis of breast cancer cells (Yeung et al., 2011).

Feng et al. (2020) showed that IRF2BP2 overexpression not only inhibited cell growth and tumor formation but also induced apoptosis, as indicated by the elevated expression of cleaved PARP in HCC cell lines, suggesting that this protein plays the role of tumor suppressor in these cells (Feng et al., 2020). A recent study reported that IRF2BP2 overexpression significantly suppressed cell death in lipopolysaccharide (LPS)-challenged mice, which was indicated by the reduced expression of proapoptotic Bax and cleaved caspase 3 and increased the expression of antiapoptotic Bcl2 (Li et al., 2020).

## IRF2BP2 in Angiogenesis

Angiogenesis is an important factor in the progression of cancer, and in the absence of vascular support, tumor may become necrotic or and tumor cells may even become apoptotic. Angiogenesis is stimulated when tumor tissues require nutrients and oxygen and is necessary for the metastatic spread of cancerous tissue (Nishida et al., 2006). Angiogenesis is regulated by both activator and inhibitor molecules, including vascular EGF (VEGF), a powerful angiogenic agent in neoplastic tissues, as well as in normal tissues (Liekens et al., 2001).

A study developed by Stewart’s group identified IRF2BP2 as a novel coactivator of VEGFA expression in muscle cells. It was demonstrated that IRF2BP2 participates in a transcriptional complex that includes TEAD4/VGLL4 proteins and coactivates VEGFA promoter expression. Interestingly, the co-expression of IRF2BP2 and TEAD1 was sufficient to coactivate the VEGFA promoter in myoblasts. Moreover, it was observed that IRF2BP2 protein levels are increased in both ischemic skeletal and cardiac muscle, showing that IRF2BP2 plays an important role in the regulation of tissue angiogenesis (Teng et al., 2010).

## IRF2BP2 in the Regulation of Inflammation

The inflammatory process is a cofactor in carcinogenesis. The inflammatory microenvironment of tumors is characterized by the presence of host leucocytes both in the supporting stroma and in tumor areas. Tumor-infiltrating lymphocytes may contribute to cancer growth and metastasis and to the immunosuppression associated with malignant disease (Coussens and Werb, 2002).

Several studies have demonstrated the importance of IRF2BP2 in regulating the inflammatory process. Feng et al. (2020) demonstrated that hepatocyte-specific IRF2BP2-deficient mouse lines (IRF2BP2-HKO cells) showed hepatic steatosis, insulin resistance, and inflammation. Real-time PCR analysis of liver tissues in high-fat diet groups revealed that the mRNA levels of inflammatory cytokines, such as TNF, were higher in the IRF2BP2-HKO mice. In contrast to these results, IRF2BP2 overexpression significantly alleviated hepatic and systemic inflammation. Moreover, complete depletion of IRF2BP2 in human hepatocytes (IRF2BP2-KO cells) led to an increase in the mRNA levels of inflammatory cytokines. This study provided evidence that IRF2BP2 regulates hepatocyte inflammation by repressing activating transcription factor 3 (ATF3) gene transcription through physical DNA binding; however, the mechanisms by which IRF2BP2 represses ATF3 gene expression are not yet clear (Feng et al., 2020).

A study developed by Chen et al. (2015) demonstrated the important function of IRF2BP2 in macrophage-mediated inflammation. They generated animals with IRF2BP2-deficient macrophages and observed the participation of IRF2BP2 in the polarization of macrophages. IRF2BP2 overexpression is involved in the differentiation of M2 type macrophages through the regulation of the expression of the anti-inflammatory transcription factor Krüppel-like factor 2 (KLF2). In addition, mice with IRF2BP2-deficient macrophages developed severe atherosclerosis (Chen et al., 2015). In a more recent study from the same group, it was observed that the loss of IRF2BP2 in microglia was associated with the reduced activation of many M2 anti-inflammatory markers and increased expression of inflammatory cytokines. IRF2BP2 is necessary to mediate the anti-inflammatory and protective effects of IFN-β cytokines on stroke injury (Cruz et al., 2017), and IRF2BP2-deficient microglia block the anxiolytic effect of enhanced postnatal care (EPC) by reducing inflammatory cytokine expression in the hypothalamus (Hari et al., 2017).

It is important to highlight the study performed by Li et al. (2020) that reported IRF2BP2 as a negative regulator of septic cardiomyopathy. Overexpression of IRF2BP2 in the heart inhibited NF-κB signaling and blocked the production of proinflammatory cytokines. Additionally, IRF2BP2 reduced inflammatory cell infiltration by suppressing the accumulation of CD11b-positive cells in the heart after lipopolysaccharide (LPS) treatment (Li et al., 2020).

## IRF2BP2 in Cell Migration and Invasion

Cell migration is required for many biological processes, such as tissue repair and regeneration. Aberrant regulation of cell migration drives the progression of many diseases, including cancer invasion and metastasis. Malignant cancer cells utilize their intrinsic migratory ability to invade adjacent tissues and the vasculature and ultimately to metastasize (Yamaguchi and Condeelis, 2007).

Manjur et al. (2019) used RNA-seq data and wound healing assays to demonstrate that the silencing of IRF2BP2 increased the migration of HEK293 cells upon dexamethasone treatment (Manjur et al., 2019). On the basis of transwell and wound healing assays, another study showed that IRF2BP2 overexpression inhibited the migration of HCC cells. On the other hand, knocking out IRF2BP2 promoted cell migration and invasion. Together, these studies showed that IRF2BP2 may play an important role in controlling cell mobility (Feng et al., 2020).

## IRF2BP2 in the Regulation of Cell Differentiation

Cell differentiation constitutes a complex biological process that regulates the expression of a large number of genes linked to the control of cell proliferation. Carcinogenesis, in turn, is characterized by the production of cell clones with genetic and epigenetic changes, which mainly result in loss of control over cell differentiation and proliferation (Chen and Dent, 2014).

Stadhouders et al. (2015) demonstrated that IRF2BP2 appears to be important for erythropoiesis and erythroid gene regulation *in vitro* and *in vivo*. IRF2BP2 interacts with the transcription factor ETO2 and favors its repressive activity in erythroid progenitor cells. The ETO2-IRF2BP2 axis recruits the NCOR1/SMRT corepressor complex and suppresses the expression of the vast majority of erythroid genes and pathways involved in terminal differentiation. The functional relevance of IRF2BP2 repressive activity was confirmed *in vivo*. It was observed that homozygous IRF2BP2-deficient mice have a lethal phenotype, dying during pregnancy or in the first weeks of life due to severe growth retardation (Stadhouders et al., 2015).

Interferon regulatory factor 2-binding protein 2 was identified as a novel determinant in the fate of neutrophil-macrophage progenitor cells, favoring macrophage development during definitive myelopoiesis in zebrafish. IRF2BP2-deficient embryos showed a significant decrease in neutrophil markers and an increase in monocyte and macrophage markers. IRF2BP2 acts as a direct target of C/ebpa and represses pu.1 gene transcription by binding directly to its promoter, and the repressive activity of IRF2BP2 is dependent on SUMOylation (Wang et al., 2020a).

A study developed by Kim et al. (2019) revealed that IRF2BP2 controls osteoclast and osteoblast differentiation *via* KLF2. IRF2BP2 overexpression increased KLF2 expression, significantly inhibited osteoclast differentiation and promoted osteoblast differentiation in bone marrow-derived macrophage cells (BMMs). Taken together, these results suggest that the IRF2BP2/KLF2 axis regulates bone homeostasis and may be a potential therapeutic target for various bone diseases (Kim et al., 2019).

## IRF2BP2 in Cancer Immunomodulation

Immune checkpoints are known to be essential regulators of immune responses. These factors are related to tumor cell escape mechanisms, for example, through the skewed regulation of the programmed death-ligand 1/programmed cell death 1 (PD-L1/PD-1) axis (Garcia-Diaz et al., 2017); this immune checkpoint promotes a break in T lymphocyte activation, leading to immune response suppression and culminating in the impairment of cytokine production and lymphocyte cytolytic activity (Garcia-Diaz et al., 2017). PD-L1 overexpression is a relevant tumor feature in several tumor types. PD-L1 is a transmembrane protein that is regulated transcriptionally by IRF-1 in response to high IFN-γ levels. IRF-1 is an IRF-2 antagonist, and these transcription factors are described as PD-L1 positive and negative regulators, respectively (Dorand et al., 2016). The tumor microenvironment is an inflammatory milieu that contains cytokines, such as the TNF and IFN family members IL-6 and IL-17. This inflammatory microenvironment contributes to tumor initiation and establishment (Zhang et al., 2018). In addition, IFN-γ in the microenvironment induces IRF-1-mediated PD-L1 transcription, establishing a critical tumor immune escape mechanism through the PD-L1/PD-1 axis (Dorand et al., 2016).

Dorand et al. (2016) observed an IRF-2 and IRF2BP2 increase in CD4 T cells deficient in Cdk5, a serine–threonine kinase that is highly active in many cancers and important in immune evasion of tumor cells. An IRF2BP2 increase is followed by hyperphosphorylation, which is essential for its nuclear localization and function (Dorand et al., 2016). Furthermore, the same study identified IRF2BP2 as an indirect PD-L1 repressor *via* IRF-2 increases and posttranslational IRF2BP2 modification, even in the presence of persistent IFN-γ stimuli (Dorand et al., 2016). Moreover, the relationship between PD-L1 and IRF2BP2 in cancer cells was also investigated by Soliman et al. (2014), who associated low IRF2BP2 levels with high PD-L1 expression in breast cancer cells (Soliman et al., 2014). Although many studies have demonstrated that IRF2BP2 participates in PD-L1 regulation, the mechanism involved in this pathway remains unclear. It must be further investigated due to its potential relevance and putative applicability in cancer immunotherapy; for example, the induction of PD-L1 downregulation may control tumor development.

Additionally, VGLL4 was recently associated with IRF2BP2 in PD-L1 expression modulation. VGLL4 is a transcriptional suppressor that competes with YAP to bind TEADs, thereby restraining YAP-induced overgrowth and tumorigenesis. Furthermore, VGLL4 promotes IRF2BP2 stability, and *Vgll4* deficiency is followed by decreased PD-L1 expression (Wu et al., 2019). IRF2BP2 absence prevents the *Cd274* transcription induced by IFN-γ, and consequently, its protein levels are reduced. Thus, VGLL4 appears to be a PD-L1 expression regulator and plays a key role in the VGLL4 and YAP association in tumor immunity modulation. Therefore, the authors suggest that IFN-γ stimulation promotes IRF-2 release from the PD-L1 promoter to favor IRF2BP2-IRF-2 binding, allowing PD-L1 expression (Wu et al., 2019). However, the mechanisms of this regulation need to be further investigated to improve cancer therapy. For instance, VGLL4 is used as an inhibition target to promote indirect *Cd274* transcription repression through IRF2BP2 stabilization.

Furthermore, another protein associated with several tumors is the transcription factor NFAT. The NFAT family is characterized as inducers of several cellular processes, including lymphocyte development, activation, and differentiation (Rao et al., 1997). NFAT1-5 expression was identified at the mRNA and protein levels in several cell types in different tumors (Mancini and Toker, 2009). The NFAT family is expressed in several cancer cell types and plays roles in survival, invasive migration, angiogenesis, and inflammatory microenvironment maintenance (Ryeom et al., 2008). In breast cancer cells, a member of the NFAT family, NFAT1, is ubiquitinated by the E3 ubiquitin ligase murine double minute 2 (MDM2) downstream of Akt and GSK-3 signaling (Wang et al., 2020b). Activated NFAT1 promotes the migration and invasion of breast cancer cells *in vitro* (Jauliac et al., 2002). Therefore, our group has identified IRF2BP2 as an NFAT1 protein interaction partner. Ectopic IRF2BP2 expression in CD4 T cells represses IL-2 and IL-4-induced NFAT1-mediated transcription; however, although these data suggest IRF2BP2 is an important immune modulator, the mechanisms involved in these processes are unclear (Carneiro et al., 2011).

Based on the importance of NFAT1 in the immune response and tumor cells, it is crucial to elucidate the repressor mechanisms mediated by IRF2BP2. Our group also demonstrated that IRF2BP2 overexpression in CD4 T lymphocytes repressed STAT5 phosphorylation and the expression of the IL-2 high-affinity receptor 𝛼-chain (CD25). Moreover, IRF2BP2 downregulates CD69 expression. Taken together, these data demonstrated that IRF2BP2 regulating CD4 T cell activation by repressing IL-2 signaling, and restraining CD4 T cells clonal expansion (Secca et al., 2016).

To investigate new antigens capable of inducing a proper and efficient immune response and cell transformation control, Blotta et al. (2009) performed a screening of a recombinant cDNA expression library from the serum of patients with monoclonal gammopathy of undetermined significance (MGUS). IRF2BP2 was identified among these newly tracked antigens. Nonetheless, the mechanisms involved in lining IRF2BP2 expression to the malignancy of plasma cells during the evolution from MGUS to multiple myeloma (MM) were not explored (Blotta et al., 2009).

**Figure 2:**
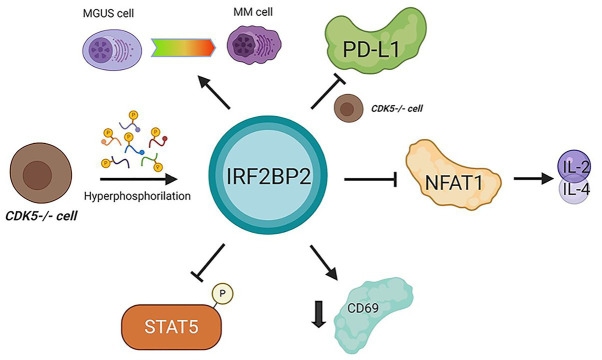
Interferon regulatory factor 2-binding protein 2 in cancer immunomodulation. IRF2BP2 was described as being involved in monoclonal gammopathy of undetermined significance (MGUS) transformation to multiple myeloma (MM) cells. When CDK5 is depleted programmed death-ligand 1 (PD-L1) is inhibited. In CD4 T lymphocytes, IRF2BP2 is associated with restraining the IL-2 and IL-4 transcription mediated by NFAT1, decreasing the CD69 marker levels and inhibiting STAT5 phosphorylation. CDK5 depletion leads to IRF2BP2 hyperphosphorylation. MGUS, monoclonal gammopathy of undetermined significance; MM, multiple myeloma.

These data are driving substantial demand for investigations of IRF2BP2 in cancer and immunological contexts. We know that IRF2BP2 represses NFAT1 transcriptional activity, inhibits STAT5 phosphorylation, downregulates CD69, and indirectly re-primes PD-L1 transcription in Cdk5-deficient T CD4 cells, and plays other roles (Figure 2). Together, these findings indicate roles for IRF2BP2 in the cancer immune response and cancer response, and this association must be elucidated. Here, we instigate a provocative and needed challenge to investigate this relationship more deeply.

## Role of IRF2BP2 and Its Genetic Variations in the Development of Tumor

The identification of molecular mechanisms and gene expression profiles necessary for tumor development and maintenance are fundamental and contribute to the diagnosis, prognosis and possible therapeutic interventions of cancer.

**Figure 3:**
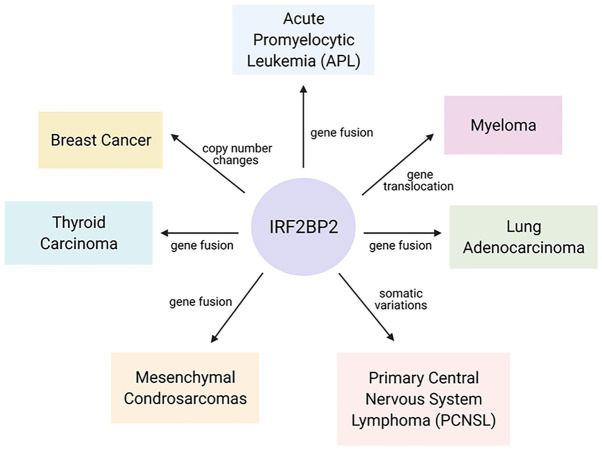
Interferon regulatory factor 2-binding protein 2 in the malignancy of hematopoietic and solid tumors. Gene fusion, copy number changes and mutations in IRF2BP2 coding sequences were found in different kinds of cancer and are linked to an increased probability of cancer development.

Some studies have reported the participation of IRF2BP2 in the development of different kinds of cancer (Figure 3) through gene fusion, copy number variations and mutations. To date, some reports have published studies on a novel fusion gene important for acute promyelocytic leukemia (APL) development. APL is characterized by the fusion of RARA with PML; however, a rare variant of this fusion gene, IRF2BP2-RARA, was identified in APL patients. This fusion gene caused some unusual clinical features and influenced the response to conventional treatment with all-trans-retinoic acid (ATRA; Yin et al., 2015; Shimomura et al., 2016; Jovanovic et al., 2017; Mazharuddin et al., 2018; Liu et al., 2019).

Furthermore, a newly discovered fusion of genes encoding IRF2BP2 and the transcription factor CDX1 was reported in mesenchymal chondrosarcomas. This in-frame t(1;5)(q42;q32) fusion results in an IRF2BP2-CDX1 translocation between exon 1 of the IRF2BP2 gene on chromosome 1 and intron 1 of the CDX1 gene on chromosome 5; however, the biological implications of this predicted fusion gene remain unknown (Nyquist et al., 2012).

Fusion involving the IRF2BP2 gene has also been identified in lung adenocarcinoma. Through next-generation sequencing, tumor samples were subjected to mutational profiling, and an in-frame gene rearrangement involving IRF2BP2 exon 1 and NTRK1 exons 8–16 was identified. No other oncogenic alterations were identified, supporting the idea that the IRF2BP2-NTRK1 fusion gene acts as a potent oncogenic driver (Wang et al., 2019). In thyroid carcinoma, a kinase involving IRF2BP2-NTRK1 fusion was found in one patient (Chu et al., 2020).

A study by He et al. (2020) identified IRF2BP2 as a novel triple-negative breast cancer (TNBC) candidate driver gene by integrating DNA copy number changes and mRNA expression omics data. The biological function of IRF2BP2 in controlling TNBC cell proliferation was assessed by siRNA-mediated loss-of-function screening of two TNBC cell lines (BT549 and SUM149T cells), and the results showed that IRF2BP2 is important in promoting proliferation in TNBC cells. In addition, copy number gain (CNG) recurrence of IRF2BP2 was found in 10/20 TNBC cell lines, indicating that this gene exhibited CNG/amplification and high expression in breast tumors (He et al., 2020).

Mutations in the IRF2BP2 gene have also been identified in primary central nervous system lymphoma (PCNSL). IRF2BP2 was sequenced to determine the coding exons by pyrosequencing, and somatic variations (nonsense mutations) were found in 14% of the PCNSL cases (Bruno et al., 2014). Alterations in the IRF2BP2 gene were predicted to be a potential prognostic markers and therapeutic targets in the treatment and management of multiple myeloma (Ni et al., 2012). A chromosomal gain was identified at region 1q42.3, which encodes IRF2BP2, in six of eight MM patients with the BCL1/JH t(11;14) translocation gene, with high penetrance (75%). Although the expression of IRF2BP2 at the mRNA level was not investigated, chromosome 1q gain is frequently associated with poor prognosis for myeloma patients (Ni et al., 2012).

## Conclusion and Perspectives

Initially, IRF2BP2 was described as a nuclear protein that interacts with IRF-2, although it has IRF-2-independent functions. Recently, IRF2BP2 has emerged as an important novel transcriptional cofactor in different biological systems, acting as a positive and negative regulator of gene expression. Although the IRF2BP2 protein is an important regulator of gene expression, little is known about how it exerts control. The details of IRF2BP2 regulation and the mechanism by which this protein regulates gene expression will be essential to understand its role in different biological processes. Recent data suggest the participation of IRF2BP2 in controlling various hallmarks of oncogenesis, acting as a tumor suppressor or oncogene depending on the cellular context and show the association of the expression of IRF2BP2 in hematopoietic and solid tumors. The details of IRF2BP2 regulation and the mechanisms involved in the relationship between the expression of IRF2BP2 and cell malignancy are not yet fully understood, and it is of great interest to understand how it influences the transcription of important genes for tumor development.

## Author Contributions

TP and BP reviewed the literature and wrote the manuscript. JV wrote and reviewed the manuscript. All authors contributed to the article and approved the submitted version.

### Conflict of Interest

The authors declare that the research was conducted in the absence of any commercial or financial relationships that could be construed as a potential conflict of interest.

## References

[ref1] Barysch S. V. Stankovic-Valentin N. Miedema T. Karaca S. Doppel J. Achour T. N.. (2021). Transient deSUMOylation of IRF2BP proteins controls early transcription in EGFR signaling. EMBO Rep. 22:e49651. 10.15252/embr.201949651, PMID: 33480129 PMC7926235

[ref2] Blotta S. Tassone P. Prabhala R. H. Tagliaferri P. Cervi D. Amin S.. (2009). Identification of novel antigens with induced immune response in monoclonal gammopathy of undetermined significance. Blood 114, 3276–3284. 10.1182/blood-2009-04-219436, PMID: 19587378 PMC2759650

[ref3] Bruno A. Boisselier B. Labreche K. Marie Y. Polivka M. Jouvet A.. (2014). Mutational analysis of primary central nervous system lymphoma. Oncotarget 5, 5065–5075. 10.18632/oncotarget.2080, PMID: 24970810 PMC4148122

[ref4] Caglar H. Avci C. (2020). Alterations of cell cycle genes in cancer: unmasking the role of cancer stem cells. Mol. Biol. Rep. 47, 3065–3076. 10.1007/s11033-020-05341-6, PMID: 32112300

[ref5] Carneiro F. R. Ramalho-Oliveira R. Mognol G. P. Viola J. P. (2011). Interferon regulatory factor 2 binding protein 2 is a new NFAT1 partner and represses its transcriptional activity. Mol. Cell. Biol. 31, 2889–2901. 10.1128/MCB.00974-10, PMID: 21576369 PMC3133407

[ref6] Chen T. Dent S. (2014). Chromatin modifiers: regulators of cellular differentiation. Nat. Rev. Genet. 15, 93–106. 10.1038/nrg3607, PMID: 24366184 PMC3999985

[ref7] Chen H. H. Keyhanian K. Zhou X. Vilmundarson R. O. Almontashiri N. A. M. Cruz S. A.. (2015). IRF2BP2 reduces macrophage inflammation and susceptibility to atherosclerosis. Circ. Res. 117, 671–683. 10.1161/CIRCRESAHA.114.305777, PMID: 26195219

[ref8] Childs K. S. Goodbourn S. (2003). Identification of novel co-repressor molecules for interferon regulatory factor-2. Nucleic Acids Res. 31, 3016–3026. 10.1093/nar/gkg431, PMID: 12799427 PMC162335

[ref9] Chu Y. H. Wirth L. J. Farahani A. A. Nosé V. Faquin W. C. Dias-Santagata D.. (2020). Clinicopathologic features of kinase fusion-related thyroid carcinomas: an integrative analysis with molecular characterization. Mod. Pathol. 33, 2458–2472. 10.1038/s41379-020-0638-5, PMID: 32737449 PMC7688509

[ref10] Coussens L. Werb Z. (2002). Inflammation and cancer. Nature 420, 860–867. 10.1038/nature01322, PMID: 12490959 PMC2803035

[ref11] Cruz S. A. Hari A. Qin Z. Couture P. Huang H. Lagace D. C.. (2017). Loss of IRF2BP2 in microglia increases inflammation and functional deficits after focal ischemic brain injury. Front. Cell. Neurosci. 11:201. 10.3389/fncel.2017.00201, PMID: 28769762 PMC5515910

[ref12] Dorand R. D. Nthale J. Myers J. T. Barkauskas D. S. Avril S. Chirieleison S. M.. (2016). Cdk5 disruption attenuates tumor PD-L1 expression and promotes antitumor immunity. Science 353, 399–403. 10.1126/science.aae0477, PMID: 27463676 PMC5051664

[ref13] Elmore S. (2007). Apoptosis: a review of programmed cell death. Toxicol. Pathology 35, 495–516. 10.1080/01926230701320337, PMID: 17562483 PMC2117903

[ref14] Fagerberg L. Hallström B. M. Oksvold P. Kampf C. Djureinovic D. Odeberg J.. (2014). Analysis of the human tissue-specific expression by genome-wide integration of transcriptomics and antibody-based proteomics. Mol. Cell. Proteomics 13, 397–406. 10.1074/mcp.M113.035600, PMID: 24309898 PMC3916642

[ref15] Feng X. Lu T. Li J. Yang R. Hu L. Ye Y.. (2020). The tumor suppressor interferon regulatory factor 2 binding protein 2 regulates hippo pathway in liver cancer by a feedback loop in mice. Hepatology 71, 1988–2004. 10.1002/hep.30961, PMID: 31538665

[ref16] Garcia-Diaz A. Shin D. S. Moreno B. H. Saco J. Escuin-Ordinas H. Rodriguez G. A.. (2017). Interferon receptor signaling pathways regulating PD-L1 and PD-L2 expression. Cell Rep. 19, 1189–1201. 10.1016/j.celrep.2017.04.031, PMID: 28494868 PMC6420824

[ref17] Hari A. Cruz S. A. Qin Z. Couture P. Vilmundarson R. O. Huang H.. (2017). IRF2BP2-deficient microglia block the anxiolytic effect of enhanced postnatal care. Sci. Rep. 7:9836. 10.1038/s41598-017-10349-3, PMID: 28852125 PMC5575313

[ref18] He J. McLaughlin R. van der Beek L. Canisius S. Wessels L. Smid M.. (2020). Integrative analysis of genomic amplification-dependent expression and loss-of-function screen identifies ASAP1 as a driver gene in triple-negative breast cancer progression. Oncogene 39, 4118–4131. 10.1038/s41388-020-1279-3, PMID: 32235890 PMC7220851

[ref19] Jauliac S. López-Rodriguez C. Shaw L. M. Brown L. F. Rao A. Toker A. (2002). The role of NFAT transcription factors in integrin-mediated carcinoma invasion. Nat. Cell Biol. 4, 540–544. 10.1038/ncb816, PMID: 12080349

[ref20] Jovanovic J. V. Chillón M. C. Vincent-Fabert C. Dillon R. Voisset E. Gutiérrez N. C.. (2017). The cryptic IRF2BP2-RARA fusion transforms hematopoietic stem/progenitor cells and induces retinoid-sensitive acute promyelocytic leukemia. Leukemia 31, 747–751. 10.1038/leu.2016.338, PMID: 27872498

[ref21] Kim I. Kim J. H. Kim K. Seong S. Kim N. (2019). The IRF2BP2-KLF2 axis regulates osteoclast and osteoblast differentiation. BMB Rep. 52, 469–474. 10.5483/BMBRep.2019.52.7.104, PMID: 31186082 PMC6675247

[ref22] Koeppel M. van Heeringen S. J. Smeenk L. Navis A. C. Jansen-Megens E. M. Lohrum M. (2009). The novel p53 target gene IRF2BP2 participates in cell survival during the p53 stress response. Nucleic Acids Res. 37, 322–335. 10.1093/nar/gkn940, PMID: 19042971 PMC2632907

[ref23] Li T. Luo Q. He L. Li D. Li Q. Wang C.. (2020). Interferon regulatory factor-2 binding protein 2 ameliorates sepsis-induced cardiomyopathy via AMPK-mediated anti-inflammation and anti-apoptosis. Inflammation 43, 1464–1475. 10.1007/s10753-020-01224-x, PMID: 32239393

[ref24] Liekens S. de Clercq E. Neyts J. (2001). Angiogenesis: regulators and clinical applications. Biochem. Pharmacol. 61, 253–270. 10.1016/S0006-2952(00)00529-3, PMID: 11172729

[ref25] Liu Y. Xu F. Hu H. Wen J. Su J. Zhou Q.. (2019). A rare case of acute promyelocytic leukemia with IRF2BP2-RARA fusion; and literature review. Onco. Targets. Ther. 12, 6157–6163. 10.2147/OTT.S217622, PMID: 31447564 PMC6684484

[ref26] Malumbres M. Barbacid M. (2001). To cycle or not to cycle: a critical decision in cancer. Nat. Rev. Cancer 1, 222–231. 10.1038/35106065, PMID: 11902577

[ref27] Mancini M. Toker A. (2009). NFAT proteins: emerging roles in cancer progression. Nat. Rev. Cancer 9, 810–820. 10.1038/nrc2735, PMID: 19851316 PMC2866640

[ref28] Manjur K. Lempiäinen J. K. Malinen M. Palvimo J. J. Niskanena E. A. (2019). IRF2BP2 modulates the crosstalk between glucocorticoid and TNF signaling. J. Steroid Biochem. Mol. Biol. 192:105382. 10.1016/j.jsbmb.2019.105382, PMID: 31145973

[ref29] Mazharuddin S. Chattopadhyay A. Levy M. Y. Redner R. L. (2018). IRF2BP2-RARA t(1;17)(q42.3;q21.2) APL blasts differentiate in response to all-trans retinoic acid. Leuk. Lymphoma 59, 2246–2249. 10.1080/10428194.2017.1421761, PMID: 29350080 PMC6162987

[ref30] Ni I. Ching N. G. Meng C. K. Zakaria Z. (2012). Translocation t(11;14) (q13;q32) and genomic imbalances in multi-ethnic multiple myeloma patients: a Malaysian study. Hematol. Rep. 4:e19. 10.4081/hr.2012.e19, PMID: 23087808 PMC3475941

[ref31] Nishida N. Yano H. Nishida T. Kamura T. Kojiro M. (2006). Angiogenesis in cancer. Vasc. Health Risk Manag. 2, 213–219. 10.2147/vhrm.2006.2.3.213, PMID: 17326328 PMC1993983

[ref32] Nyquist K. B. Panagopoulos I. Thorsen J. Haugom L. Gorunova L. Bjerkehagen B.. (2012). Whole-transcriptome sequencing identifies novel IRF2BP2-CDX1 fusion gene brought about by translocation t(1;5)(q42;q32) in mesenchymal chondrosarcoma. PLoS One 7:e49705. 10.1371/journal.pone.0049705, PMID: 23185413 PMC3504151

[ref33] Rao A. Luo C. Hogan P. G. (1997). Transcription factors of the NFAT family: regulation and function. Annu. Rev. Immunol. 15, 707–747. 10.1146/annurev.immunol.15.1.707, PMID: 9143705

[ref34] Ryeom S. Baek K.-H. Rioth M. J. (2008). Targeted deletion of the calcineurin inhibitor DSCR1 suppresses tumor growth. Cancer Cell 13, 420–431. 10.1016/j.ccr.2008.02.018, PMID: 18455125

[ref35] Secca C. Faget D. V. Hanschke S. C. Carneiro M. S. Bonamino M. H. De Araujo-Souza P. S.. (2016). IRF2BP2 transcriptional repressor restrains naive CD4 T cell activation and clonal expansion induced by TCR triggering. J. Leukoc. Biol. 100, 1081–1091. 10.1189/jlb.2A0815-368R, PMID: 27286791 PMC6608065

[ref36] Shimomura Y. Mitsui H. Yamashita Y. Kamae T. Kanai A. Matsui H.. (2016). New variant of acute promyelocytic leukemia with IRF2BP2-RARA fusion. Cancer Sci. 107, 1165–1168. 10.1111/cas.12970, PMID: 27193600 PMC4982591

[ref37] Soliman H. Khalil F. Antonia S. (2014). PD-L1 expression is increased in a subset of basal type breast cancer cells. PLoS One 9:e88557. 10.1371/journal.pone.0088557, PMID: 24551119 PMC3925108

[ref38] Stadhouders R. Cico A. Stephen T. Thongjuea S. Kolovos P. Baymaz I.. (2015). Control of developmentally primed erythroid genes by combinatorial co-repressor actions. Nat. Commun. 6:8893. 10.1038/ncomms9893, PMID: 26593974 PMC4673834

[ref39] Teng A. C. Al-Montashiri N. A. Cheng B. L. Lou P. Ozmizrak P. Chen H. H.. (2011). Identification of a phosphorylation-dependent nuclear localization motif in interferon refulatory factor 2 binding protein 2. PLoS One 6:e24100. 10.1371/journal.pone.0024100, PMID: 21887377 PMC3162591

[ref40] Teng A. C. Kuratis D. Deeke S. A. Ahmadi A. Dugan S. G. Cheng B. L. M.. (2010). IRF2BP2 is a skeletal and cardiac muscle-enriched ischemia-inducible activator of VEGFA. FASEB J. 24, 4825–4834. 10.1096/fj.10-167049 20702774

[ref41] Tinnikov A. A. Yeung K. T. Das S. Samuels H. H. (2009). Identification of a novel pathway that selectively modulates apoptosis of breast cancer cells. Cancer Res. 69, 1375–1382. 10.1158/0008-5472.CAN-08-2896, PMID: 19190336 PMC4264605

[ref42] Wang B. Gao Y. Huang Y. Ou Q. Fang T. Tang C.. (2019). Durable clinical response to crizotinib in IRF2BP2-NTRK1 non-small-cell lung cancer. Clin. Lung Cancer 20, e233–e237. 10.1016/j.cllc.2018.12.017, PMID: 30691963

[ref43] Wang L. Gao S. Wang H. Xue C. Liu X. Yuan H.. (2020a). Interferon regulatory factor 2 binding protein 2b regulates neutrophil versus macrophage fate during zebrafish definitive myelopoiesis. Haematologica 105, 325–337. 10.3324/haematol.2019.217596 31123027 PMC7012491

[ref44] Wang W. Zafar A. Rajaei M. Zhang R. (2020b). Two birds with one stone: NFAT1-MDM2 dual inhibitors for cancer therapy. Cell 9:1176. 10.3390/cells9051176, PMID: 32397368 PMC7291050

[ref45] Wu A. Wu Q. Deng Y. Liu Y. Lu J. Liu L.. (2019). Loss of VGLL4 suppresses tumor PD-L1 expression and immune evasion. EMBO J. 38:e99506. 10.15252/embj.201899506, PMID: 30396996 PMC6589543

[ref46] Yamaguchi H. Condeelis J. (2007). Regulation of the actin cytoskeleton in cancer cell migration and invasion. Biochim. Biophys. Acta 1773, 642–652. 10.1016/j.bbamcr.2006.07.001 16926057 PMC4266238

[ref47] Yao Y. Wang Y. Li L. Xiang X. Li J. Chen J.. (2019). Down-regulation of interferon regulatory factor 2 binding protein 2 suppresses gastric cancer progression by negatively regulating connective tissue growth factor. J. Cell. Mol. Med. 23, 8076–8089. 10.1111/jcmm.14677, PMID: 31559693 PMC6851004

[ref48] Yeung K. T. Das S. Zhang J. Lomniczi A. Ojeda S. R. Xu C. F.. (2011). A novel transcription complex that selectively modulates apoptosis of breast cancer cells through regulation of FASTKD2. Mol. Cell. Biol. 31, 2287–2298. 10.1128/MCB.01381-10, PMID: 21444724 PMC3133243

[ref49] Yin C. Jain N. Mehrotra M. Zhagn J. Protopopov A. Zuo Z.. (2015). Identification of a novel fusion gene, IRF2BP2-RARA, in acute promyelocytic leukemia. J. Natl. Compr. Cancer Netw. 13, 19–22. 10.6004/jnccn.2015.0005, PMID: 25583766 PMC5843191

[ref50] Zhang S. Yang X. Wang L. Zhang C. (2018). Interplay between inflammatory tumor microenvironment and cancer stem cells. Oncol. Lett. 16, 679–686. 10.3892/ol.2018.8716, PMID: 29963133 PMC6019921

